# Condition-dependent trade-offs maintain honest signalling

**DOI:** 10.1098/rsos.220335

**Published:** 2022-10-05

**Authors:** Szabolcs Számadó, Flóra Samu, Károly Takács

**Affiliations:** ^1^ Department of Sociology and Communication, Budapest University of Technology and Economics, Egry J. u. 1. H-1111, Budapest, Hungary; ^2^ CSS-RECENS, CSS-RECENS, Centre for Social Sciences, Tóth Kálmán u. 4, H-1097, Budapest, Hungary; ^3^ Agglomeration and Social Networks Lendület Research Group, Centre for Economic-and Regional Studies, CSS-RECENS, Centre for Social Sciences, Tóth Kálmán u. 4, H-1097, Budapest, Hungary; ^4^ Corvinus University of Budapest, Doctoral School of Sociology, Fővám tér. 8, H-1093, Budapest, Hungary; ^5^ The Institute for Analytical Sociology, Linköping University, S-601 74 Norrköping, Sweden

**Keywords:** communication, honest signalling, handicap principle, costly signalling, condition-dependent trade-offs

## Abstract

How and why animals and humans signal reliably is a key issue in biology and social sciences that needs to be understood to explain the evolution of communication. In situations in which the receiver needs to differentiate between low- and high-quality signallers, once a ruling paradigm, the Handicap Principle has claimed that honest signals have to be costly to produce. Subsequent game theoretical models, however, highlighted that honest signals are not necessarily costly. Honesty is maintained by the potential cost of cheating: by the difference in the marginal benefit to marginal cost for low versus high-quality signallers; i.e. by differential trade-offs. Owing to the difficulties of manipulating signal costs and benefits, there is lack of empirical tests of these predictions. We present the results of a laboratory decision-making experiment with human participants to test the role of equilibrium signal cost and signalling trade-offs for the development of honest communication. We found that the trade-off manipulation had a much higher influence on the reliability of communication than the manipulation of the equilibrium cost of signal. Contrary to the predictions of the Handicap Principle, negative production cost promoted honesty at a very high level in the differential trade-off condition.

## 1. Introduction

To understand the evolution of communication in animals and humans, it is essential to find out why signals of senders should be trusted by receivers. Honest signalling is challenging to explain if some senders, for instance in the absence of relevant quality, have an interest to deceive the receivers. The receivers face the problem of how to differentiate between senders with and without the relevant quality if both send the same signal. Yet signals are plentiful in both nature and human interactions and many signals are informative to the receiver. For example, in birds of paradise, males display complex dance moves to attract their mate. Why did such elaborated signals evolve and what do they signal? Why should a female pick a male with an extraordinary display?

Claiming a solution to the puzzle, the Handicap Principle proposed that to be honest, signals need to be costly to produce, and consequently, they have to function as handicaps that only high-quality individuals can bear [1–3]. For instance, the peacock's train can serve as an honest signal of peacock's quality because it is costly to produce [1]. Zahavi's idea remained contested [4,5] until Grafen [2] published an analytical model in which he claimed to show that honest biological signals have to function as handicaps (‘if we see a character which does signal quality, then it must be a handicap’; [2], p. 521). The Handicap Principle remained highly influential in biology despite criticism through the years [4–16] as researchers still interpret their results in light of the Handicap Principle (e.g. [17–19]). Zahavi's idea was adopted in other disciplines as well, such as anthropology [20–22]. It inspired studies on human mate choice [23–25], generosity [26,27], consumer behaviour [28], big game hunting [20,21], trophy hunting [29], risk taking in young man [30,31], criminal behaviour [32], citizen behaviour [33]; blood donations [34], over-consumption of resources [35] and religious rituals [36–38], just to name a few examples (see [22] for review). While recent work is more critical of the Handicap Principle (see [39–41]), the idea remained an influential explanation of human behaviour.

Grafen's claim, however, was not substantiated as Grafen's model is not a handicap model at all and Grafen's main handicap results are unsupported by his own model (see [16] for more detail). On the one hand, Grafen [2] correctly identified the need of differential marginal costs as a mechanism that can maintain honest signalling. On the other hand, this insight was not novel as it is an insight learnt also by the theory of signalling in economics (see [42,43]). Spence however, has never proposed that individuals must pay a handicap cost in order for the signal to be honest [41]. Subsequent game theoretical models have made it clear that it is not the equilibrium cost of the signal that maintains the honesty of communication but the difference of *marginal costs* to *marginal benefits* by signaller type [9,44–46]. That is, honesty can only be guaranteed if the marginal benefits of giving the signal are higher than the marginal costs of transmission for individuals with the inherent quality, while the opposite holds for individuals without the quality. This means that honest signals need not be costly to produce at the equilibrium not even under conflict of interest [9,12,13,45]. Highly costly signals could be sent by signallers with the relevant quality and these signals, such as earning a university diploma, will be trusted by receivers because the difference in the benefits and production costs for individuals with the relative quality is higher than for individuals without the quality. However, cost-free or even negative cost signals can be honest and evolutionarily stable as well [9,12,44,45,47,48]. Telling about a good grade earned at school to the parents is cost-free or even self-rewarding and can expected to be believed because the marginal benefits to marginal costs are higher for those who actually earned a good grade compared to those who did not. Cost-free and self-rewarding signals are not rare in nature, for instance, singing to attract mates or enjoying the sun on the highest panoramic cliff to signal dominance.

Note, that many signalling games in economics have taken a different route (compared to biology) and investigated the relevance of ‘cheap talk’ models. Cheap talk is defined as a message where ‘players' messages have no direct pay-off implications [49]. In a seminal model of the field Crawford & Sobel [50] were able to show that such cheap talk can be informative as long as the preferences of the signaller and receiver are similar. This inspired a long line of research asking what kind of equilibria are possible in cheap talk games and out of these what can be observed in humans [51–56]. Our approach differs from this line of experiments by investigating the role of signal costs and trade-offs.

The closest to our approach is a recent experiment by Rubin *et al*. [57] in which they investigated the possibility of hybrid equilibria arising in which signals are not fully but partially informative in a signal-response game with conflict of interest in a laboratory setting. They found that partially informative communication has developed. As a main difference to our investigation, in their experiment, they contrasted the hybrid equilibrium manipulation with a simple control condition in which interests were aligned to allow fully informative communication. Hence, they did not systematically test if (partially) honest signalling emerged owing to costly signalling or owing to signalling trade-offs. All in all, experimental evidence is still lacking whether signals used by humans need to be costly to produce to be honest, or not. To fill this gap, we conducted a laboratory experiment where human participants played a signalling game.

## 2. Signalling games and terminology

**Figure 1.**
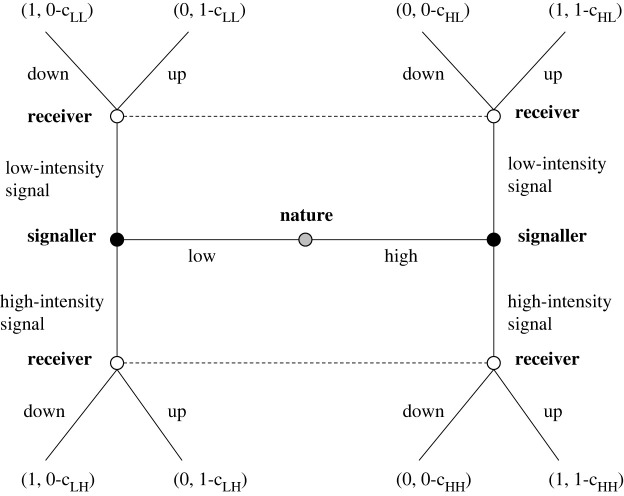
The signalling game with conflict of interest. A differential cost model when the cost is condition-dependent. After nature randomly divides the roles (S and R) and the types of signallers (high- and low-quality S), players make sequential decisions. First, S can send two different signals (low-intensity signal, high-intensity signal) to get the resource from R. After this, R receives the signal and decides whether to provide the resource to S or not. At the end of each round players get feedback on their success. Both high- and low-quality S wins when they get the resource from R, but for R only the dedication of the resource to a high-quality S and its protection from low-quality S generate successful outcomes (see outcomes in brackets at the ends of the decision tree where the first element refers to R and the second to S). The reward for success is indicated simply as 1 in the figure, in the experiment it meant a pay-off of HUF 1200. In the figure c_LL_, c_LH_, c_HL_ and c_HH_ indicate the different cost of signals according to the type of signaller.

In general, signalling games have been fruitful to model various dyadic situations of communication and to provide insights about the conditions of honesty and reliability of messages. The signal-response game describes the interaction of a signaller (S) and a receiver (R) under the assumption of asymmetrical information, namely that the signaller knows its own quality relevant to the receiver (high versus low-quality), while the receiver does not (figure 1). The signaller may or may not give a signal (high versus low-intensity signal), finally the receiver may or may not transfer the resource to the signaller. The hidden type of the signaller is important here since R gets the highest pay-off if it gives the resource to a high-quality S instead of a low-quality one. At the same time, however, both signallers are interested in receiving the resource from R (there is a conflict of interest). Since R does not have information on the type of S, R is trying to predict the type of the signaller from the signal while a low-quality S is trying to conceal it, because otherwise, with ‘honest’ communication, they would not receive the resource from R.

The focus of signalling games is to investigate the equilibrium conditions of *honest* communication where the receiver can tell the unseen quality of the signaller from the use of (different) signals, i.e. there is a correlation between quality and signal intensity. At the honest equilibrium, the signal will be *informative*, because the receiver can infer from the intensity of the signal the quality of the sender. Hence, the receiver will *trust* the signal, i.e. R will transfer the resource to signaller using a high-intensity signal and R will reject signallers using a low-intensity signal. Thus, as a result of honesty and trust, the receiver will be able to accurately transfer the resource to high-quality signallers (*accuracy*). An equilibrium strategy pair is defined as a set of strategies for the signaller and for the receiver such that it does not benefit to deviate unilaterally for any of the players. The equilibrium cost of signals is the signal cost observed at equilibrium. Since at the honest equilibrium only high-quality S will use the high-intensity signal, the observed equilibrium cost is the cost of high-intensity signals for high-quality type signallers. It is natural to believe that in any signalling system it takes time to realize the equilibrium strategies and hence signal-response games are typically played repeatedly in the laboratory setting.

The honest equilibrium is often called as ‘separating’ as signaller types are separated by their use of signal [46], which leads to a consequence that signals identify the type of the signaller. There can also be other types of equilibria such as ‘pooling’, where some or all of the signaller types are using the same signal [58] or ‘hybrid’, where some types can be mixing different signals with a given probability [59]. Note that these later equilibria (pooling and hybrid) will not be entirely honest, however, they need not be (and probably will not be) entirely uninformative either.

**Figure 2.**
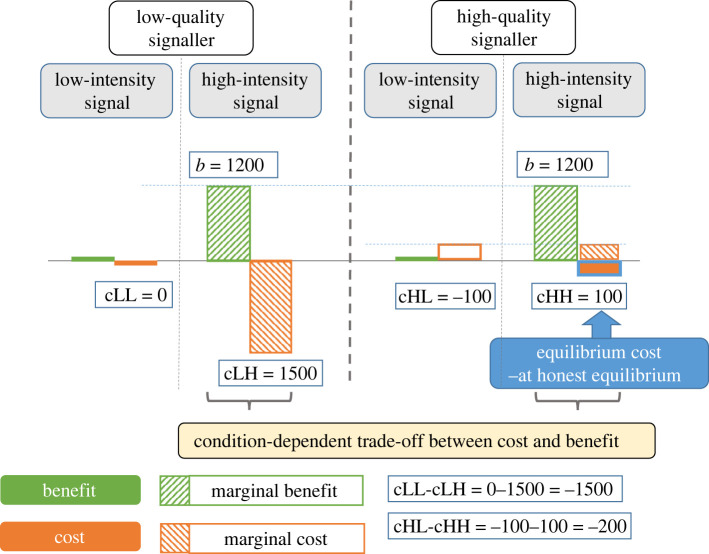
The relationship between various costs and the concept of conditional trade-off. Pay-offs correspond to treatment no. 9 in table 1. The equilibrium cost at honest equilibrium for the high-quality signaller is indicated by a filled orange bar in the right column. The condition-dependent trade-off between cost and benefit is displayed as the relative difference between the green and orange bars for low-quality signallers on the left and high-quality signallers on the right of the figure.

Last but not least figure 2 describes the relationship between various costs and the concept of trade-off. Equilibrium cost describes the cost paid by high-quality signallers for the use of a high-intensity signal (i.e. it describes a state). Marginal cost describes the (cost) difference between switching from one type of signal to the other (i.e. it describes change in one function). Finally, a trade-off describes a specific relationship of two functions: increasing the value of one function is not possible without decreasing the other.

## 3. Manipulations and hypotheses

**Figure 3.**
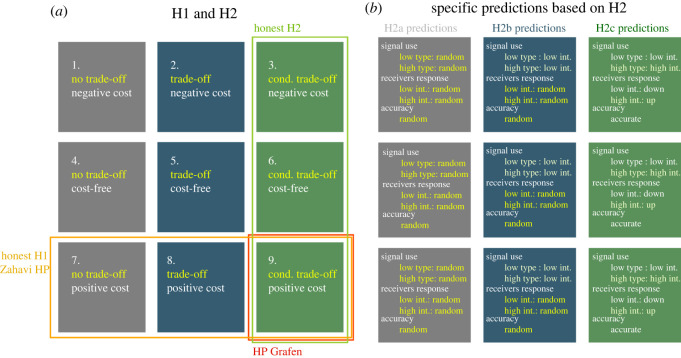
General and specific predictions (H1 and H2). (*a*) The design matrix with nine manipulations. Rows: equilibrium signals with negative cost, cost-free, positive cost. Columns: signals without trade-off, with trade-off and with conditional trade-off. H1: prediction of Zahavi's Handicap Principle; honesty is maintained by positive equilibrium cost. H2: prediction of recent game theoretical models; honesty is maintained by condition-dependent trade-offs. Grafen: intersection of H1 and H2. (*b*) Specific predictions derived for each trade-off treatment based on H2.

In our experiments, by using a two-factorial experimental design we varied both the production cost of the signal for high-quality signallers and the presence of signalling trade-offs (table 1). Signallers could either have high- or low-quality. First, in the signalling cost manipulation (between rows in figure 3*a*) we varied whether (i) high-quality signallers had to pay to use the high-intensity signal; (ii) high-quality signallers could use the high-intensity signal for free; or (iii) high-quality signallers received a benefit (payment) for using the high-intensity signal. Second, in the trade-off manipulation (between columns in figure 3*a*), we tested whether the absence, the presence or the differentiation of difference between the costs of the two signals by the quality of signallers influence the emergence of honest communication. Accordingly, we had three trade-off conditions: (i) no trade-off, where the cost of low- and high-intensity signal was the same regardless of the quality of the signaller; (ii) trade-off condition, where sending the high-intensity signal was costlier for both low- and high-quality signallers in the same way (i.e. the marginal cost of sending a high-intensity signal was the same for all signallers); and (iii) condition-dependent trade-off condition, where the cost of sending a high-intensity signal was relatively costlier for low-quality signallers than for high-quality signallers (i.e. the marginal cost of sending a high-intensity signal was higher for low-quality signallers than for high-quality signallers). Note that taking the high-intensity signal is never cheaper than taking the low-intensity signal.

**Table 1.** Treatment conditions. (The table shows the costs low- and high-quality signallers have to pay (in HUF) for using low-intensity or high-intensity signals (c_LL_ for low-intensity signal, c_LH_ for high-intensity signal for a low-quality signaller, c_HL_ for low-intensity signal, c_HH_ for high-intensity signal for a high-quality signaller). Costs are indicated with positive numbers. Negative numbers indicate negative costs (i.e. positive rewards for choosing the given signal). First, treatments differed in whether the use of the high-intensity signal is costly, cost-free, or profitable for high-quality signallers. Second, treatments also differed in whether there was no trade-off between sending the low-intensity and high-intensity signal (c_LH_−c_LL_ = c_HH_−c_HL_ = 0), there was a fixed trade-off between the two signals (c_LH_−c_LL_ = c_HH_−c_HL_ = 1500), or the trade-off between the two signals was condition-dependent (c_LH_−c_LL_ = 1500; c_HH_−c_HL_ = 200).)

treatment no.	trade-off	low-quality signaller		high-quality signaller		expectation by the Handicap Principle	expectation by game theoretical models
		low-intensity signal	high-intensity signal	low-intensity signal	high-intensity signal		
		c_LL_	c_LH_	c_HL_	c_HH_		
1.	no	0	0	−100	−100	dishonest	dishonest
2.	yes	0	1500	−1600	−100	dishonest	dishonest
3.	condition-dep.	0	1500	−300	−100	dishonest	honest
4.	no	0	0	0	0	dishonest	dishonest
5.	yes	0	1500	−1500	0	dishonest	dishonest
6.	condition-dep.	0	1500	−200	0	dishonest	honest
7.	no	0	0	100	100	honest	dishonest
8.	yes	0	1500	−1400	100	honest	dishonest
9.	condition-dep.	0	1500	−100	100	honest	honest

We derive two conflicting predictions based on previous literature. The Handicap Principle [1,3] predicts that signals will be honest only if high-quality signallers have to pay a wasteful (positive) cost for sending the high-intensity signal. By contrast, more recent game theoretical models [9,44–46] predict that signals will be honest under the condition of differential (condition-dependent) trade-offs irrespective of the cost paid at the equilibrium by honest signallers.

H1: the signal cost paid by high-quality signallers maintains the honesty of signals.

H2: the honesty of communication is maintained by signalling trade-offs, in such a way that for honest communication it has to be fulfilled that the marginal cost of high-intensity signal compared to the low-intensity signal for high-quality signallers must be cheaper than for low-quality signallers.

H2 means that the difference in costs between the high-intensity and the low-intensity signal has to be smaller for high-quality signallers than for low-quality signallers. H2 does not exclude the possibility that costs are negative, hence H1 and H2 are in conflict (as seen in figure 3*a*). The implications of Grafen [2] could be seen as the intersection of H1 and H2 (bottom right cell in figure 3*a*).

We need to elaborate more detailed predictions implied by H2 (figure 3*a*,*b* for the experimental design and comparison of predictions). In the condition where the costs of the two signals are equal for both types of signallers—or in other words where there is no trade-off between the signals—high-quality signallers cannot gain anything by selecting one particular signal with the same cost, because costs are the same also for low-quality signallers. Therefore, honest communication cannot evolve since there are no such individual strategies from which it is not beneficial for either party to move towards another action.

H2a: in the absence of trade-off: (i) the signallers are expected to select randomly from the two available signals, therefore (ii) receivers respond also randomly to the signals they receive; and (iii) the accuracy of decisions made by receivers are not better than picking randomly without signals.

In the condition in which there is a fixed trade-off between the two signals, both low- and high-quality signallers are expected to send the same signal. Costs in the experiment are configured in a way that the marginal cost of using the high-intensity signal exceeds the benefit of the resource (see detailed costs in table 1). As a consequence, signallers in either the low or high condition would be motivated to send the low-intensity signal. Hence, we expect a pooling equilibrium, in which both signallers send the low-intensity signal and therefore, the receiver is unable to predict the type of the signaller.

H2b: if both types of signallers have the same trade-off between the two signals, then (i) both signaller types will use the same signal, and (ii) receivers decide randomly on the allocation of the source; and (iii) the accuracy of decisions made by receivers are not better off than picking randomly without signals.

Pay-offs under the condition-dependent trade-off treatment have been chosen to fit the conditions of separating equilibrium (signaller types separate by sending different signals, i.e. signalling is honest), derived by discrete game theoretical models [45]. That is, the potential benefit of signalling out-weights the potential cost for high-condition signallers, while it is not the case for low-quality signallers. High-quality signallers will switch to the high-intensity signal in order to acquire the resources from the receiver since in this case, the marginal cost of moving to this signal is smaller than the marginal benefit of it. Low-quality signallers will stick to choosing the low-intensity signal, and therefore honest communication develops.

H2c: if the two signaller types have a different trade-off between the use of two signals in such a way that high-quality signallers have larger marginal benefits for choosing the high-intensity signal than low-quality signallers, then (i) the two types will use different signals depending on their type, thus (ii) the receivers will be able to determine the type of signallers correctly and respond differently; and (iii) the accuracy of decisions made by receivers are better off than picking randomly without signals, i.e. they can allocate the resources according to their interest.

Predictions are summarized in figure 3*b*.

## 4. Methods

### 4.1. Experimental set-up

Participants played a simple 2 × 2 signalling game in a computer laboratory. The experiment took place at the Corvinus University of Budapest (CUB) in Hungary between 25 January 2018 and 15 January 2019. Participants were regular or corresponding students. In total, 12 sessions were organized, involving different numbers of participants (groups of 12, 16 and 20) and 196 students participated in the experiment (118 women out of 196 students). A mix of within and between subject design was applied: within a session, each group played three selected treatments of the nine possible conditions. The selected games varied from session to session and each condition was played as the first, second and third game. Since the order of games and the number of participants may affect the speed of learning dynamics in the game, we control for these factors with statistical methods during the analysis. During the experiment, participants were seated randomly in front of the computers, thus participants took part in the experiment anonymously. Computers were connected to a local network with the help of the software z-Tree [60]. The description of the game was displayed on participants' screens (see the electronic supplementary material, S5). Instructions were provided on paper as well. At the end of the experiment, participants received a show-up fee of HUF 1000 and the pay-off of one randomly selected round.

### 4.2. Experimental task

In each round, as a first step, participants were randomly divided into groups of four containing two signallers (S) and two receivers (R). In the experiment, we called them player X and Y, respectively. Moreover, we used an unbiased signalling game, where the two types of signallers (high- and low-quality S) were assigned randomly by the computer. In a group of 20, for instance, this meant 10 receivers: five high-quality and five low-quality signallers. Groups and roles were changed round-by-round. In the experiment instead of using ‘high’ and ‘low quality’, neutral categories were used to differentiate between the two types. High-quality signallers were presented as blue players and low-quality signallers were depicted in yellow. Everyone was only aware of their own type; they did not know each other's type.

First, player X (either blue or yellow) had to choose a signal and send it to player Y in order to get the resource. High- and low-intensity signals were also replaced by neutral pairs of signs (‘)(’, ‘∼’; ‘//’, ‘O’, ‘[]’, ‘<>’). These characters and their costs (that could have been a benefit in the negative costs treatment) in the given condition were displayed. While player X in the blue or yellow condition had selected one of the two displayed characters, player Y was waiting.

In the next step, receivers (player Y) decided whether they would give the resource after a signal (the character sent by player X) was seen. Signallers succeeded if they received the resource, but receivers' success depended on the (hidden) type of the signaller (high-quality S was preferred to low-quality S). After these steps, participants learned the type of the sender, the signal they sent and the success of their decision. The game was played for 20 rounds [52,54]. In the case of a successful decision, they received HUF 1200. In addition to this, the cost of the signal influenced the final payment (table 1).

In the interests of clarity, we show an example of the calculation:

Example 1: Y can select from the following signs: (i) using the <> sign reduces Y's pay-off by HUF 300; (ii) using the [] sign increases Y's pay-off by HUF 100. Y decided to use the <> sign and X gave him the resource. In this case, the pay-off of Y will be 1200–300 = 900.

## 5. Analytical approach

As main outcome measures of our analysis, we defined honesty, trust and accuracy, and we examine these outcomes in each treatment. First, we report simple averages and correlation statistics and then we provide in-depth regression analysis to test and control for more design elements of the experiment (for instance treatment and time). The latter allows us to deal with the imbalances between experimental sessions as well (slight differences in group size; order of play; the use of different characters as signals). In the following, we describe how we specify each outcome in each analysis.

**Figure 4.**
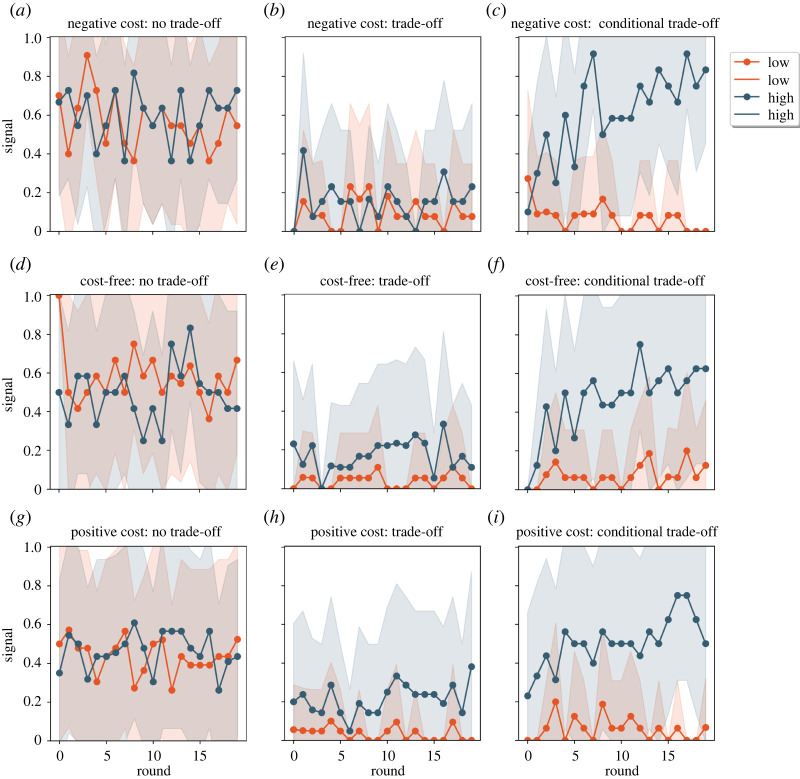
Signaller decisions by rounds and by the nine treatments. Left column: no trade-off manipulation; middle column: trade-off manipulation; right column: conditional trade-off manipulation. Upper row: negative signal cost for high-quality signallers, middle row: cost-free for high-quality signallers, bottom row: positive signal cost for high-quality signallers. ‘Signaller decision’ panels: dark blue line shows the proportion of high-quality signallers using high-intensity signals, the orange/red line shows the proportion of low-quality signallers using high-intensity signals. At the honest equilibrium all high-quality signallers should use the high-intensity signal and none of the low-quality ones (dark blue at 1; orange/red at zero). Dots show the average per round, overlay shows one standard deviation.

**Figure 5.**
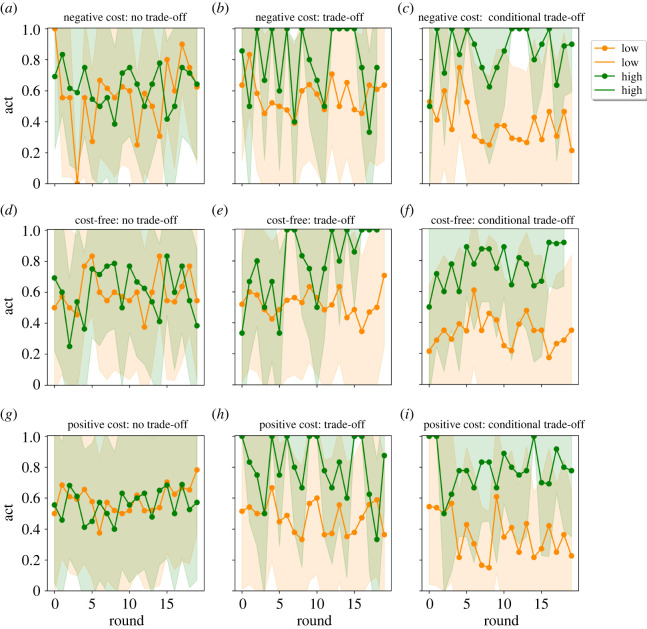
Receiver decisions by rounds and by the nine treatments. Left column: no trade-off manipulation; middle column: trade-off manipulation, right column: conditional trade-off manipulation. Upper row: negative signal cost for high-quality signallers, middle row: cost-free for high-quality signallers, bottom row: positive signal cost for high-quality signallers. ‘Receiver decision’ panels: green line shows the proportion of receivers giving the resource to high-intensity signals, the orange line is for giving the resource to low-intensity signals. At the honest equilibrium receivers should always give the resource in response to high-intensity signals and they should never give to low-intensity ones (green line at 1; orange at 0). Dots show the average per round, overlay shows one standard deviation.

**Figure 6.**
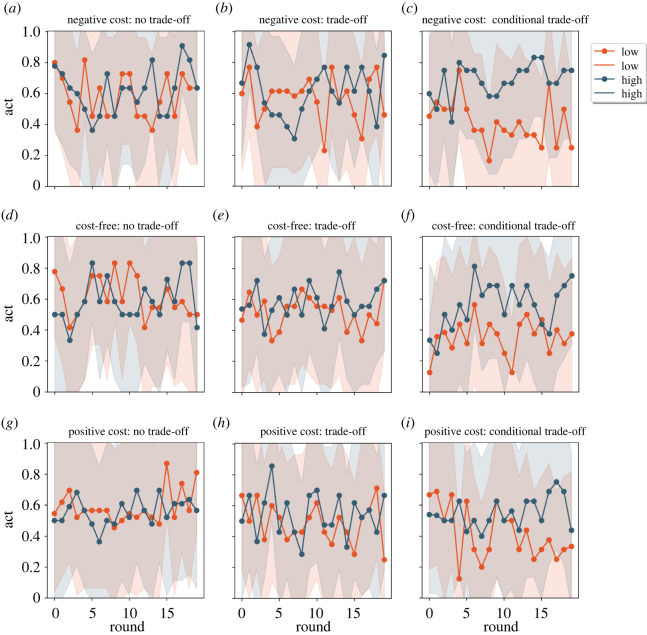
Receivers’ accuracy by rounds and by the nine treatments. Left-hand column: no trade-off manipulations, middle-colum: trade-off manipulations, right-hand column: conditional trade-off manipulations. Upper row: negative signal cost for high-quality signallers, middle row: cost-free for high-quality signallers, bottom row: positive signal cost for high-quality signallers. ‘Receiver accuracy’ panels: dark blue and orange/red lines show the proportion of high- and low-quality signallers receiving the resource respectively. At the honest equilibrium, receivers can find out the quality of the signaller from the signal, thus they should donate the resource only to high-quality signallers and never to low-quality ones (dark blue at 1; orange/red at zero). Dots show the average per round, overlay shows one standard deviation.

After showing descriptive statistics of the general tendencies regarding the three outcomes visually in figures 4–6, we start testing each theoretical prediction by calculating Pearson correlation coefficients for each round in each treatment. The honesty score shows the correlation between signaller type and signal sent; it can vary between −1 (high-quality signallers send low-intensity signals and low-quality types send high-intensity signals) and 1 (high-quality signallers send high-intensity signals and low-quality types send low-intensity signals), where 0 means no correlation (i.e. signalling is random and hence the signal is uninformative). The trust score shows the correlation between the receiver's decision and the signal received. It is calculated as the honesty score, where 0 means that the receivers are not relying on the signal in their decisions (i.e. they are giving out the resource randomly). The accuracy of the decision by the receiver is measured as the correlation between the allocation of the resource and the quality of the signaller: it varies between −1 (meaning that the receiver allocates the resource to low-quality signallers against their interest) and 1 (meaning that the receiver correctly allocates the resource to high quality signallers), where 0 means that receiver allocates the resource randomly. It is important to note that in the ‘no trade-off’ manipulation both signals can develop to be honest because high-intensity signals are not determined by the costs of the signals (both signals are free or have the same costs).

Multi-level mixed-effects logistic regressions are used for analysing binary outcomes. Players made binary choices about sending one of the two signals, and when they decided whether to give the resource at the sight of a signal. Success is also a binary outcome if giving resource to high-quality signallers and refusing it from low-quality players is considered as 1 and any other decision as 0. Specifically, by testing honesty we estimate whether the probability of selecting low-intensity signals (dependent variable) by high-quality signallers (independent variable) is higher than doing so by low-quality signallers. Negative effect indicates honesty, meaning that compared to low-quality signallers the probability of sending low-intensity signals is lower if decision maker is a high-quality signaller. Trust is also translated into probabilities: the probability of refusing the resource (dependent variable) after observing the low-intensity signal (independent variable) compared to observing high-intensity signals. Positive effects indicate more trust. Accuracy is specified as the probability of refusing giving the resource but now the condition is the hidden type of the signaller. These models test whether the probability of refusing the resource is higher when a low-quality signaller asks for it. Again, positive parameter estimations imply accuracy.

In these models, we test the temporal dimension of outcomes by including an interaction effect between the above-mentioned independent variables (honesty, trust, accuracy) and round of the games (see models with a label ‘b’ in the electronic supplementary material, tables S17–S21). The same sign of the interaction term and the parameter estimation of the main independent variable (the type of the signaller and the signal) indicates that honesty, trust and accuracy are intensifying over time. In addition to this in the models, we control for the equilibrium cost manipulations to show that results are independent of it. Lastly, we use control variables to fix imbalances between the experimental sessions. In the text we report the estimated coefficients, and odds ratios as well to indicate the size of the effects.

## 6. Results

In this section three outputs will be examined: (i) to what extent are signals with different intensity separated by the signallers' condition (i.e. whether signals are honest; honesty score), (ii) how resource allocation is made according to which signal was seen (i.e. whether the receiver responds selectively to the signal; trust score), and (iii) the overall degree of coordination between response and condition, to what extent has the quality of the signallers been successfully determined by the receivers (i.e. whether the receivers were successful in making an optimal decision; accuracy). In the following, we provide more precise definitions of these outcomes in accordance with the methodology we use.

### 6.1. Aggregate results

We look at the aggregate results first (see figures 4–6), and then we run more complex models to test the outlined outputs after we control for other variables (signs used in the game, rounds, order of the games number of participants; see tables and detailed descriptions of the effects in the electronic supplementary material, S3 and S4).

Figures 4–6 show the timeline of the experiment as a function of the experimental manipulations (no trade-off, trade-off, conditional trade-off) where three figures (4–6 respectively) each show: signal use, receiver's decision and receiver's accuracy. Accordingly, honesty, trust and accuracy can be inferred implicitly from the separation of the two lines that are intended to represent the two types of signallers (in figures 4 and 6) and signals (figure 5) respectively. Lines approaching the value of 1 show the increasing proportion of high-intensity signals (figure 4) and resource allocation (figure 5). Please note that in the ‘no trade-off’ manipulation both signals can function as a high-intensity signal (i.e. one signal is denoted arbitrarily as the high-intensity type for the plot). Before we address this question in a more detailed assessment, first take a brief look at the simple signal selection in this and in the other trade-off treatments, where costs imply the type of the signal and therefore also honesty and trust.

There is no association between signal use and signaller's type because both signals are cost-free or have the same positive or negative cost in the ‘no trade-off’ manipulation, meaning that it is not determined which signal would attract receivers' positive decision. Accordingly, signallers try to assign meaning to both signals, therefore we do not see any difference between the two lines (figure 4, first column: blue-red). There is some separation of signaller types under the ‘trade-off’ manipulation because a higher proportion of high-quality signallers use high-intensity signals (figure 4, second column: blue-red). Finally, there is a clear separation of signaller types under the ‘conditional trade-off’ manipulation (figure 4, third column: blue-red). The receiver's decisions seem to follow this trend (figure 5, second columns: green-orange); yet an increase in accuracy is only observed in the last manipulation (figure 6, third columns: blue-red).

**Figure 7.**
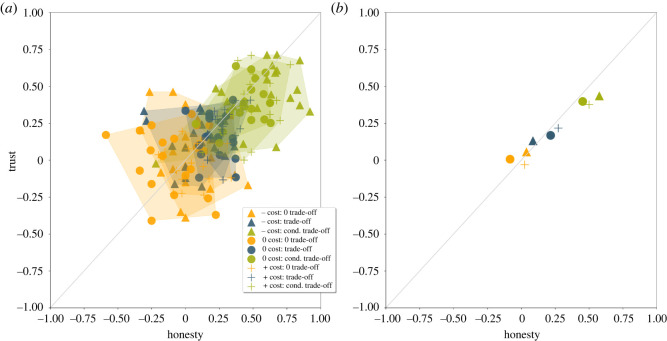
Honesty and trust scores as a function of experimental manipulations. (*a*) Honesty and trust scores by rounds and by manipulations, polygons show the rounds belonging to the same manipulation; and (*b*) the honesty and trust score of each manipulation. Orange: no trade-off, blue: trade-off, green: conditional trade-off; circle: negative cost, triangle: no cost, plus sign: positive cost.

Plotting honesty and trust together also separates the manipulations (figure 7). In figure 7 honesty and trust are expressed as Pearson correlation coefficients between signallers' state and signals, and signals and actions of the receivers, respectively. Figure 7*a* shows the honesty and trust scores by manipulations per rounds, while figure 7*b* displays the average of honesty and trust scores per manipulations. Average scores indicate that the trust placed into signals is proportional to the honesty of signals. Honesty and trust scores in the ‘conditional trade-off’ manipulation—irrespectively of the signal cost—are the highest, indicating that signals in this manipulation were the most honest and most trusted. Even in this condition, the values are far from perfect correlations (i.e. far from the top right corner).

### 6.2. Hypothesis testing

Next, we go through the results in the order of the hypotheses. Pearson's chi-squared tests are shown to assess our main hypotheses H1 and H2.

H1. We see similar patterns by the manipulation of equilibrium signalling cost (compare results between rows in figures 4 and 5), which suggests that the signalling cost paid has no effect on honest communication. We cannot see a significant difference between negative, cost-free and positive cost conditions either in honesty (*χ*^2^ = 4.78, *p* = 0.09; table 2) or in trust (*χ*^2^ = 0.77, *p =* 0.68; table 2) and consequently, the same is true for accuracy (*χ*^2^ = 3.62, *p* = 0.16; table 2).

**Table 2.** Honesty, trust and accuracy by treatment conditions with statistical tests for significant differences. (* p < 0.05, ** p < 0.01, *** p < 0.001.)

	honesty (%)		trust (%)		accuracy (%)	
	dishonest	honest	inconsequential	meaningful	failure	success
negative eq. cost	40.63	59.38	44.44	55.56	45.21	54.79
zero eq. cost	43.04	56.96	44.67	55.33	47.28	52.72
positive eq. cost	39.66	60.34	43.37	56.63	48.46	51.54
Pearson's chi-squared test	4.78	0.77	3.62			
no trade-off	50.92	49.08	49.61	50.39	51.84	48.16
trade-off	44.09	55.91	48.94	51.06	48.22	51.78
conditional trade-off	29.03	70.97	33.64	66.36	41.93	58.07
Pearson's chi-squared test	174.09***		116.64***		33.61***	

H2. The honesty of signals, however, is influenced by signalling trade-offs. Figures 4 and 5 show that there are observable differences in both signallers' and receivers’ decisions based on trade-off manipulations (i.e. between columns). Statistical tests confirm these insights: sending signals corresponding to players' status (honesty) shows variation along trade-off treatments (*χ*^2^ = 174.09, *p* < 0.001; table 2). The meaning of the two signals (trust) (*χ*^2^ = 174.09116.64, *p* < 0.001; table 2) and the overall coordination between response and condition (accuracy) diverge as well (*χ*^2^ = 174.0933.61, *p* < 0.001; table 2). In the following, we discuss each treatment in the trade-off manipulation separately by presenting results of regression analyses, where we could handle imbalances between experimental sessions such as number of players, the order of the games, signs used in the experiment.

Mixed-effects logistic regressions are used to test our hypotheses H2a, H2b and H2c (see detailed results in the electronic supplementary material, S3 and S4).

H2a. ‘No trade-off’: different types of signallers used the two signals with the same probability in treatments where there was no difference in the cost of using the two signals (no trade-off) (see the non-significant effect of signaller's state on sending separating signals in model 1a, electronic supplementary material, table S17). Moreover, receivers did not trust one signal more than the other (see non-significant effect of the signal on receiver's decision in model 1a, electronic supplementary material, table S18). At the same time, accuracy has improved over time, because high-quality signallers were 3.05% more likely to receive the resource in each subsequent round (exp(−0.031)), while low-quality signallers were 1.05 times (exp(0.048)) more likely to be rejected by the receiver than high-quality signallers in each round (see round and signaller's state–round interaction effects in model 1b, electronic supplementary material, table S19). The overall accuracy was 48.16% in this treatment (table 2), which is not different from random (*t*_3038_ = 1.402, *p* = 0.08). To resolve the apparent contradiction behind accuracy in the absence of honesty and trust, we carried out an analysis on the local conventions that may have developed within each experimental group in the no trade-off manipulation. We found that in half of the groups high-quality signallers used one signal in a higher proportion than low-quality signallers, while the other signal was used by them with a higher probability in the other half of the experimental groups (electronic supplementary material, figure S1). We re-coded these signals as high-intensity signals (that were more frequently used by high-quality signallers than low-quality signallers) and labelled the other signal as a low-intensity signal. By doing so, we artificially synchronize signals and signallers in this treatment in order to investigate the efficiency of local conventions. We found that local conventions emerged—again, where the directions of these conventions was random (i.e. they randomly picked one or the other signal as ‘high-intensity’; see the electronic supplementary material, figure S1)—because, low-intensity signals are 45.12% less likely (exp(−0.600)) to be selected by high-quality signallers than by low-quality signallers (see the effect of signaller's state in model 1a, electronic supplementary material, table S20). Initial convention, however, did not intensify over time (see non-significant effect of signaller's state—round interaction in model 1b, electronic supplementary material, table S20).

Looking at the receiver's responses to these signals, we also see a difference in the extent to which the receiver gave the resource after seeing the low-intensity signal. Low-intensity signals were 1.46 times more likely (exp(0.382)) rejected by the receiver than high-intensity signals (see the effect of signal intensity in model 1a, electronic supplementary material, table S21). More interestingly, this separation has developed over time: in each round the probability of refusing the resource upon high-intensity signals decreased by 3.15% (exp(−0.032)) while, in each round, the rejection of low-intensity signals were 1.05 times more likely than the rejection of high-intensity signals (see round and signal-round interaction effect in model 1b, electronic supplementary material, table S21).

Despite the development of local conventions, in the ‘no trade-off’ treatments receivers estimated signallers' original states correctly only half of the time (48.2%) (gave the resources to high-quality S and refuse to give it to low-quality S), suggesting that despite the observed intention for separation, this treatment fails to provide accuracy that is better than random. *All in all, signals were not informative and receivers did not react selectively on a global level in the no trade-off manipulation. Local conventions, however, were still formed. The direction of these local conventions was random (in parallel to the aggregate lack of honesty and trust). These conventions were learned by receivers, and as a result, the performance of the receiver improved over time. Still, receivers could not do better than random choice when allocating the resource*.

H2b. ‘Trade-off’: in treatments with the same trade-off for both type of signallers, we see that the majority (55.91%; table 2) of signallers were using one signal. Contrary to our expectation, high-quality senders intended to separate signals as they slightly shifted from low to high-intensity signals (see the effect of signaller's state in model 2a, electronic supplementary material, table S17). More precisely, selecting low-intensity signals was 83.25 per cent less likely among high-quality signallers than among low-quality signallers (exp(−1.787)). The difference, however, does not increase over time (see signaller's state and round interaction in model 2b, electronic supplementary material, table S17). We also see a difference within receivers' decisions by signals: receivers tend to favour high-intensity signals (giving 79.2% of the resource) compared to low-intensity signals (51.6%; see the electronic supplementary material, table S12). Indeed, the regression analysis suggests that receiver's preference for high-intensity signals is almost six times higher (exp(1.780)) (see the effect of signals in model 2a, electronic supplementary material, table S18). The ratio of receiver success was slightly higher than in the previous treatment (51.78%; table 2), however, it is not different than random (*t*_4158_ = −1.306, *p* = 0.10) and we found evidence that low-quality signallers were by rounds 1.04 times more likely to be rejected compared to high-quality signallers (model 2b, electronic supplementary material, table S19). *In summary, in the trade-off condition, different signals were used by different signallers. Receivers also acted selectively, yet they did not improve over time and signals did not become informative* (i.e. *receivers could not do better than random*).

H2c. ‘Conditional trade-off’: results observed in the conditional trade-off manipulation are consistent with our expectations. Signals diverge to the greatest extent by the type of signaller in these treatments. A learning dynamic can be observed in figure 4. High-quality signallers gradually switched to the use of a signal with favourable trade-off to them—*which need not be costly* (see insignificant signaller's state—equilibrium cost interaction effect in model 3c, electronic supplementary material, table S22)—to achieve coordination. Most (approx. 65%) of high-quality signallers used the high-intensity signal in the last rounds of the game while more than 90% of low-quality signallers were stuck to the low-intensity signal. According to the regression analysis, participants, indeed, moved towards the separating equilibrium: sending low-intensity signals were 96.61% less likely among high-quality signallers compared to low-quality signallers (see the effect of signaller's state in model 3a, electronic supplementary material, table S17). As a response, 80.4% of the resource was given if receivers saw the high-intensity signal and almost two-thirds of the low-intensity signals were rejected (63.7%; see the electronic supplementary material, table S13). According to the regression analysis, low-intensity signals were 10 times more likely to be rejected than high-intensity signals (exp(2.299), see the signal's effect on receiver's decision in model 3a, electronic supplementary material, table S18). In terms of overall accuracy (58.07%, which is different than random *t*_3518_=−6.449, *p* < 0.001), refusing the resource from low-quality signallers were twice as likely (exp(0.888)) than refusing it from high-quality signallers (see figures in the last column in figure 4 and the effect of signaller's (hidden) state on the resource allocation in model 3a, electronic supplementary material, table S19). Sending low-intensity signals by high-quality signallers were 14.1% less likely round-by-round than the same action by low-quality signallers (exp(−0.152), see model 3b, electronic supplementary material, table S17). In each round refusing low-intensity signals was 1.07 times more likely than refusing high-intensity signals, (see model 3b, electronic supplementary material, table S18), in each round the probability of refusing the resource from high-quality signallers decreased by 3.63% (exp(−0.037)), while, in each round, the rejection of low-quality signallers were 1.07 times more likely than the rejection of high-quality signallers (exp(0.070) model 3b, electronic supplementary material, table S19). The intensified separation of signals, actions over time and the increasing accuracy of the resource allocation are demonstrative results of the development of honest communication. *All in all, in the conditional trade-off condition honesty, trust and accuracy improved over time, by the last rounds signals were informative, receivers mostly responded selectively, and receivers achieved a higher success than random choice*.

## 7. Discussion

How animal and human communication can be reliable is a fundamental question in biology and the social sciences. When senders and receivers have mutual interests, honest communication can develop (e.g. [6,61]). Honest communication, however, is much more difficult to establish in situations with conflict of interest, because low-quality senders could mimic high-quality senders by sending the same signal and therefore making the communication uninformative for the receiver. This study intended to test rival theoretical accounts that claimed to explain the emergence of honest communication in situations with conflict of interest by conducting experiments with human participants. The conflicting predictions tested were made by the proponents of the Handicap Principle [1–3] and by subsequent game theoretical models [9,44–46,62]. These implications have been translated into two main hypotheses. First, we tested following the Handicap Principle [13] if signalling costs for high-quality signallers alone could establish honest communication (H1). Second, we tested whether signalling trade-offs could lead to honest signalling [9,10,12,45,46] (H2). We expected that the lack of trade-offs (H2a) and trade-offs that are identical for low- and high-quality signallers (H2b) would not be sufficient for receivers to differentiate signaller types efficiently. Instead, condition-dependent trade-offs have been expected to be the guarantees of honest communication (H2c).

Our results support the last claim: informative signals emerged in our experiment under the condition-dependent trade-off condition regardless of the cost of signals for high-quality signallers. Signals with zero or even negative production cost (benefit) were honest if trade-offs were condition-dependent. An intention to separate signaller types by using different signals can be observed in the other manipulations as well (no trade-off, trade-off). Local conventions emerged in the no trade-off condition, although the direction of these conventions were random, thus at the aggregate level, no honesty or trust could be observed. There was an intention to separate in the trade-off condition as well, however, it was only mildly successful as the use of a high-intensity signal was not beneficial for either type of signaller. Thus, we can see that the intention to separate signaller types (be honest) was successful only in the condition-dependent trade-off manipulations as predicted by game theoretical models. In other words, *full-fledged honesty could not evolve in the no trade-off and trade-off treatments despite the effort of the participants to create an honest system*. Potential motivations for this intention (to be honest) will be discussed later.

Our results demonstrate that the conditions which allow the emergence of honest signalling is different and much wider from what was predicted by the Handicap Principle [1]. Equilibrium signalling cost in itself is not enough to generate an honest signalling system. Also, the region of honesty is *not constrained* to the manipulation with differential marginal cost with positive equilibrium cost as predicted by Grafen [2]. Last but not least, the highest level of honesty was achieved under the condition-dependent trade-off condition with *negative* signal cost *contrary to the prediction of the Handicap Principle*.

Not surprisingly, there is an increasing gap between the predictions of the Handicap Principle and the empirical observations. There is a growing body of literature showing the abundance of dishonest signals both in nature [63–69] and in human communication [70–74], in parallel with a growing literature showing that signals are not costly to produce [75–82]. For example, recent studies found that the flagship example of the Handicap Principle, the peacock's train, does not handicap the locomotion of peacocks [80,81]. Also, there is a growing recognition that cheap (subtle) signals can be lot more important in human communication as it would be expected based on the Handicap Principle [39,40]. Last but not least, these signalling trade-offs can be implemented between benefit functions, i.e. having a cost function *per se* is not a requirement of honest signalling. Our results thus reinforce the call for the replacement of the Handicap Principle with a Darwinian theory of signalling based on conditional signalling trade-offs [10,16] for explaining honest communication.

Overall, it is important to emphasize that no strict separating equilibria were observed in any of the treatment conditions. There was always some level of mixing indicating some randomness of decisions even among our subjects who were university students well able to grasp the structure of the simple experimental signalling game. The level of mixing, however, was much reduced under the condition-dependent trade-off manipulation. Some differentiation evolved between low- and high-type signallers in the simple trade-off manipulation. *Only the condition-dependent trade-off manipulation allowed receivers to make an informed decision* (i.e. *do better than random choice*)*.* There could be several factors influencing the level of mixing observed in the experiment. The first one is the length of the experimental sessions. As a result of a learning process, a higher differentiation is expected between signaller types in the use of signal over time. The equilibrium signaller and receiver strategies were straightforward, yet they were somewhat contra-intuitive with negative signal cost. In these later manipulations, it was naturally a longer learning process to find (or being close to) the equilibrium strategy pair. The second factor is the difference between marginal benefit and marginal cost. The higher this difference, the more obvious the marginal benefit, thus probably it is easier to find (or get closer to) the honest equilibrium (if there is any). The third factor is the roles played by the participants. Participants alternated between all three roles in our experiment (i.e. receiver, low- and high-quality signaller). There are advantages and disadvantages of this set-up. While participants might have a better understanding of the experimental set-up, they may not be playing (optimizing for) a single role. We observed an intention to separate (to signal honestly) in all manipulations, which could be owing to the fact that players alternated between all three roles, which in turn created an incentive to be honest (since both receivers and high-quality signallers are better off this way). Playing a single role might force them to optimize their actions for that single role only, thus this could remove the above effect. Alternatively, this observed effect could be owing to a more general ‘truth telling’ tendency identified in previous ‘honesty’ experiments (i.e. dice roll experiments [73], see [74] for review). The last potential factor, unaddressed in this experiment, is the potential role of individual variation. Both previous experiments on cooperation and the ‘dice roll’ experiments revealed heterogeneity of human predispositions: some of us are more cooperative [83,84] or more willing to tell the truth than others [85,86] and vice versa. If so, achieving perfect separation (i.e. a perfectly honest equilibrium) may not be possible at all. Only future experiments can tell which one of these factors is responsible for the observed level of mixing in our results.

## Data Availability

Data file from the experiment can be downloaded from: https://data.mendeley.com/datasets/dv4wn5jr2g/1. The data are provided in the electronic supplementary material [87].
